# Enhanced academic motivation in university students following a 2-week online gratitude journal intervention

**DOI:** 10.1186/s40359-021-00559-w

**Published:** 2021-05-13

**Authors:** Norberto Eiji Nawa, Noriko Yamagishi

**Affiliations:** 1 Center for Information and Neural Networks (CiNet), National Institute of Information and Communications Technology (NICT), Room 2A2, 1-4 Yamadaoka, Suita, Osaka 565-0871 Japan; 2 Graduate School of Frontiers Biosciences, Osaka University, Suita, Japan; 3 College of Global Liberal Arts, Ritsumeikan University, Ibaraki, Japan

**Keywords:** Gratitude, Positive emotion, Academic motivation, Online intervention, Gratitude journal

## Abstract

**Background:**

Past studies have associated gratitude interventions with a host of positive outcomes. However, there is a dearth of research regarding the impact such interventions have on the academic motivation of university students, thought to be a primary determinant of academic achievement and overall satisfaction with school activities. Here, we examined the effects of a 2-week online gratitude journal intervention on the academic motivation of university students.

**Methods:**

Eighty-four students were randomly assigned to either an active manipulation group (gratitude group) or a neutral control group. In the first 6 days of each week, participants in the gratitude group were asked to log in to the online system once a day and list up to five things they had felt grateful for. They were also requested to evaluate various aspects of their daily lives. Participants in the control group were only requested to perform the daily self-evaluations. Academic motivation was assessed using the Academic Motivation Scale (AMS), which conceptualizes motivation in academic settings as being composed by three different components, i.e., intrinsic motivation, extrinsic motivation and amotivation, the latter being associated with the perceived lack of contingency between actions and outcomes. Responses were collected 5 times: before group assignment (baseline), 1 week after the start of the intervention, immediately after the intervention, and at two follow-ups, 1 and 3 months after the intervention.

**Results:**

Analysis using a self-determination index derived from the AMS components showed that participants who regularly engaged with the gratitude journal task displayed significant enhancements in academic motivation. Additional analysis revealed that the enhancements were driven by decreases in the levels of amotivation. Furthermore, follow-up data showed that there were no signs that such enhancements had receded 3 months after the end of the intervention. Improvements in academic motivation were not observed among participants in the control group.

**Conclusions:**

The current results provide evidence that gratitude interventions can positively impact the academic motivation of university students. More broadly, they show that the effects extend well beyond the realm of typically assessed measures of individual well-being, and can effectively regulate a fundamental component of goal-directed behavior such as motivation.

**Supplementary Information:**

The online version contains supplementary material available at 10.1186/s40359-021-00559-w.

## Background

Positive psychology interventions have been associated with a host of positive psychological outcomes, ranging from improvements in well-being [33, 63] to the relief of depressive symptoms [33, 56, 57]. Among such interventions are gratitude interventions, i.e., activities that aim to increase the practitioner’s awareness to personal experiences associated with the emotion of gratitude, the affective response that emerges when one acknowledges and appreciates the benefits promoted by the actions of others [40].

Typical gratitude interventions activities involve having individuals write a letter expressing how grateful they are to a benefactor, e.g., [63], or asking people to regularly write about events that made them feel grateful, an activity commonly known as the gratitude journal, e.g., [23]. The majority of studies so far have examined the impact that such interventions can have in improving mood outcomes [23] and other aspects related with individual well-being, such as happiness [47, 48], life satisfaction [14], and subjective well-being [70], as well as the influence they exert on physiological variables such as blood pressure [35] and other physical health outcomes (for a review, see [6]).

Positive psychology variables like optimism have been shown to be associated with academic outcomes such as grade point average (GPA) [49]; similarly, a few studies have examined how the emotion of gratitude is linked with various aspects of a student’s life. For instance, a composite score of individual gratitude disposition based on self-reports was shown to be a better high-school GPA predictor than individual scores of materialism [30]. Middle school students who were asked to list up to five things they were grateful for, for 2 weeks, reported higher levels of satisfaction with the school experience compared to students assigned to control conditions [32]. Of interest, such differences were still detected in follow-up responses collected 3 weeks after the end of the intervention.

Several studies have shown that gratitude effects can extend beyond the modulation of psychological variables, possibly altering core processes underlying executive functions, which in effect can lead to changes in behavior. Gratitude has been hypothesized to affect behaviors by enlarging thought-action repertoires [28], much like other positive emotions as posited by the broaden-and-build theory [27]. According to that theory, in contrast with negative emotions which culminate in very specific behavioral outcomes (e.g., fight or flight), positive emotions encourage individuals to *broaden* the space of possible actions and *build* personal resources by enlarging their physical, intellectual, psychological and social reservoirs [29], enabling people to better cope with life’s many adversities and challenges. Consistent with this line of thought, studies have shown that gratitude promotes prosocial behavior, by way of greater willingness to reciprocate a favor [64] or prolonged help to benefactors and strangers as well [5]. Gratitude is also associated with enhanced self-control in the form of decreased temporal discounting, i.e., given a choice between a smaller immediate reward and a larger delayed reward, grateful people tend to regard the latter as more attractive than the former [20].

Recently, Armenta et al. [1] proposed that the experience of gratitude motivates students to engage in behaviors that lead to self-improvement, driving them to become better and more productive students. This insight is consistent with results that showed that gratitude reduces economic impatience [20], since self-improvement behaviors are typically aimed at long-term goals at the expense of immediately attainable goals. Four mechanisms were hypothesized to underlie the enhancements in self-improvement behaviors that result from the experience of gratitude [1]: increases in feelings of connectedness, increases in feelings of elevation, increases in humility as well as increases in negative states such as indebtedness and guilt. To directly examine that hypothesis, Armenta et al. [2] conducted a study in which, for 4 weeks, 9th and 10th grade students (mean age of 15.11 years old) were asked to spend 10 min every week writing a letter of gratitude to someone who helped them with their health, academics, or someone who did something kind to them. Students in the control group were asked to simply list daily activities. All participants were asked to rate the extent they felt motivated to improve themselves in the respective domain using a 1-item scale. After 4 weeks, students in the gratitude conditions displayed enhancements in self-improvement motivation; importantly, such effects were still present 3 months after the completion of the intervention [2]. Further analyses revealed that the increase in self-improvement motivation among participants in the gratitude conditions were partially mediated by enhanced feelings of connectedness, elevation and indebtedness.

Other studies have examined the relation between gratitude and different types of motivation in school contexts. King and Datu [37] looked at the relationship between the individual gratitude disposition of university students (mean age of 18.40 years old) and their motivation towards academic activities. Gratitude disposition was assessed using the Gratitude Questionnaire (GQ-6) [44] and academic motivation (autonomous and controlled) was measured using the Academic Self-regulation Questionnaire [17]. Correlational results showed that the GQ-6 scores were associated with individual levels of autonomous motivation but not controlled motivation (Study 1, [37]), which suggested that fostering gratitude among university students may lead to specific improvements in autonomous motivation.

In a subsequent gratitude intervention study (Study 3, [37]), university students (mean age of 18.13 years old) were invited to participate in a one-shot 10-min writing activity where they were asked to write a gratitude letter to someone whom they felt thankful for but had not properly expressed their gratitude. Students in the control group were asked to write about events experienced during the previous week. After the writing manipulation, all participants completed the GQ-6, the Engagement and Disaffection Scale [58], which measures different facets of engagement with classroom activities (cognitive, emotional and behavioral), and an adapted version [9] of the Academic Motivation Scale (AMS) [69]. The AMS conceptualizes academic motivation as being composed by three different components, i.e., intrinsic motivation, extrinsic motivation and amotivation, the latter being associated with the perceived lack of contingency between actions and outcomes. Academic motivation is a multi-faceted psychological construct centered on the notion that motivated students tend to perceive school-related activities as more enjoyable, and learning as a valuable and pleasant activity in itself [53]. It is thought to be a main determinant of overall student satisfaction with curricular and extra-curricular activities, and a predictor of academic achievement [59]. More importantly, academic motivation is central in initiating and maintaining goal-directed behaviors in school settings.

Results showed that after the letter writing manipulation, the GQ-6 and the cognitive and emotional engagement scores of the students in the gratitude condition were on average greater than the scores of students in the control group. However, no significant effects were detected on the AMS scores or the scores of behavioral engagement (Study 3, [37]). One possibility raised in [37] to account for the lack of motivational effects was the relative low intensity of the one-shot, 10-min gratitude letter writing activity. Gratitude interventions typically range from a few days to a few weeks and typically involve repeated and regular engagement with a gratitude activity [13, 31, 72]. Even though the dose response relationship for different types of gratitude intervention still needs to be clearly established [24], there seems to be a tendency that interventions that continuously and repeatedly engage participants are more likely to result in significant effects [6].

To address methodological gaps in the literature and provide a more conclusive test of the impact that gratitude interventions can have on the academic motivation of university students, here we examined whether regular engagement with a gratitude journal activity over the course of 2 weeks was associated with improvements in academic motivation. We implemented an online system where participants accessed the tasks that were scheduled on each one of the days of the intervention; importantly, that enabled us to verify how strictly participants complied with the experimental schedule. Participants in the active manipulation group were requested to keep a gratitude journal during the 2 weeks of the intervention; gratitude journaling is a popular activity among people proactively trying to improve their everyday life happiness [50] and thought to be more engaging than writing letters of gratitude [36]. Based on previous studies, we hypothesized that engaging with the gratitude journal would raise the students’ awareness of the academic opportunities (“blessings”) bestowed upon them, triggering a re-evaluation of motives and goals that would be expressed as improvements in academic motivation, which here was comprehensively measured using the original version of the AMS [69]. We also expected that regular engagement with the gratitude journal would exercise the students’ ability to move the focus off themselves to other people; that change would be  expressed as an improved perspective taking aptitude at the end of the intervention. Finally, we hypothesized that such transformations would   be accompanied by improvements in individual well-being, specifically, life satisfaction.

## Methods

### Participants

Participants were recruited via the social media app Twitter using an account maintained by a volunteer of the local university community solely for this purpose. A call was sent to the approximately 5,000 followers of that account on February and July of 2019; interested users were redirected to an online form, which described details of the procedures involved in the study and its schedule, the participation requirements—be aged between 20 and 30 years old, be currently enrolled as an undergraduate or graduate student in an university, be able to access the internet during the entire period of the study—and the monetary compensation they would receive upon completion of the tasks (3000 yen). Candidates who declared to meet all the requirements were asked to provide their contact information, name and gender. All candidates were then contacted via email; in accordance with the principles stated in the Declaration of Helsinki, candidates who wished to formally sign up for the study were requested to fill out and submit an online consent form. In addition, they were asked to complete the paperwork necessary to receive the monetary compensation. The study was approved by the local research ethics committee.

An a priori power analysis using G*Power [25, 26] for a two-way repeated measures ANOVA with power = 0.8, medium effect size = 0.25 and sphericity correlation value = 0.75 indicated a minimum total sample size of 34 participants. In total, 84 students who fulfilled the requirements for participation took part in the study: 48 participants during the weeks of March 2019 (17 females/31 males, mean age 22 years old, age range = 20–25) and 36 participants during the weeks of August 2019 (18 females/18 males, mean age 22 years old, age range = 20–26). On both occasions, the period in which students were actively engaged in performing the online tasks overlapped with the school break seasons (spring and summer, respectively). Because compliance with the experimental schedule was hypothesized to be a critical prerequisite for the manipulation to be effective, we set up an inclusion criterion that required that the tasks should be performed on the scheduled date on at least 2/3 of the 12 days on which a task was scheduled to be completed. Participants who did not meet this inclusion criterion were removed from the analysis. All communication with the participants, as well as the online forms and assessments were carried out in Japanese.

### Procedure

A few days prior to the start of the 2-week intervention, participants were emailed personalized kits with instructions on how to sign-in to the web-based system that was especially developed for the purposes of this study. The kit included the individual account name and initial password to be used by each participant. Participants were requested to access the system using a web browser and answer a battery of questions composed by the items of various psychological scales (see “Materials” section). After participants completed all items in the questionnaire, they were randomly assigned to either the gratitude journal group (gratitude group) or the control group. To enforce an even ratio of female and male participants across groups, male and female participants were grouped in two separate lists; each list was randomly shuffled and split into two halves. Half ± 1 of the male (female) participants were assigned to the gratitude group, while the remaining half was assigned to the control group.

Participants were instructed to log in to the online system every day during the period of the study (2 weeks). Immediately after logging in, a calendar was displayed on the screen showing the tasks that were expected to be performed on each day; by clicking on the task name (e.g., Diary), participants were redirected to a task-specific page where they inputted the requested information. No restrictions were imposed regarding the place or the time of the day participants could access the online system, the amount of time they should spend performing each task (including the gratitude journal activity), or the device that was used to connect to the system, e.g., laptop computer, smartphone, etc.

### Materials

*Gratitude disposition* The 6 items of the Gratitude Questionnaire (GQ-6) [44] were used to assess the individual disposition of experiencing the emotion of gratitude. The GQ-6 is often conceptualized as measuring trait gratitude, i.e., one’s tendency to attend and affectively respond to the role of other people in giving rise to positive outcomes that benefit the self. Respondents provide ratings to sentences such as “I have so much in life to be thankful for” ranging from 1 (*strongly disagree*) to 7 (*strongly agree*) with a neutral middle point of 4 (*neutral*). After reverse scoring the ratings for two items, a total score is computed. The GQ-6 (Japanese version) was found previously to have good internal consistency reliability (alpha = 0.92) and good 4-week test–retest reliability (r = 0.86), based on the data from 409 Japanese college students [60], with an average total score of 32.67 (time 1) and 32.49 (time 2).

The GQ-6 was collected with two goals in mind; the first was to ensure that there were no pre-existing differences at baseline between the gratitude and control groups with regard to gratitude disposition. The second goal was to verify whether there would be observable differences in trait gratitude after 2 weeks, since the GQ-6 is directly related to the target of the current manipulation.

*Life satisfaction* The 5 items of the Satisfaction With Life Scale (SWLS) [22] were used to assess the participants’ overall satisfaction with life beyond specific domains such as personal health and finances. Global satisfaction with life is thought to be one of the three major components of the construct of individual subjective well-being [21]. Respondents use a scale from 1 (*strongly disagree*) to 7 (*strongly disagree*) with a neutral middle point of 4 (*neither agree nor disagree*) to rate sentences such as “In most ways my life is close to my ideal”. The mean SWLS score computed from 176 undergraduate students in the original paper was 23.5 (SD = 6.43), and the 2-month test–retest coefficient based on data from 76 students was r = 0.82 [22].

The SWLS was collected to verify whether the current gratitude intervention would result in improvements in life satisfaction, as reported in previous studies, e.g., [70].

*Perspective Taking* The Perspective Taking Scale (PT) is one of the four scales comprising the Interpersonal Reactivity Index, a metric designed to measure individual differences regarding the multifaceted construct of empathy [15, 16], Perspective-taking refers to the ability of anticipating the behaviors of others and is conceptualized as one of the dimensions of empathy, here, the PT was used to assess one’s tendency to spontaneously adopt the point of view of others and see things from their perspective; it is made of 7 items consisting of sentences such as “I believe that there are two sides to every question and try to look at both of them”, which are rated using a scale ranging from 0 (*does not describe me well*) to 4 (*describes me very well*). Based on data collected from 1161 participants, the PT was found to have reasonable internal consistency (alpha = 0.75 for male participants and alpha = 0.78 for female participants) and the test–retest reliability was r = 0.61 for male participants and r = 0.62 for female participants (the time elapsed between the first and second administration of the questionnaire ranged from 60 to 75 days) [15].

Responses to the PT were collected to verify whether regular engagement with a gratitude journal activity would result in changes in perspective taking aptitude of participants in the gratitude group.

*Academic Motivation* The Academic Motivation Scale (AMS) [69] is a 28-item multidimensional scale developed under the tenets of the self-determination theory [54]. The AMS measure 3 types of motivation associated with academic activities based on 7 subscales [18, 67]. Intrinsic motivation is characterized by three types of behaviors that are engaged in for their own sake, because they are inherently interesting and bring enjoyment: behaviors (1) to know, (2) to accomplish things, and (3) to experience stimulation. Extrinsic motivation, in contrast, is characterized by three types of behaviors that are instrumental to achieve a goal but are not engaged for the enjoyment of the behavior itself: (4) external regulation, associated with behaviors that are directly initiated and regulated by external contingencies, such as rewards or punishments, (5) introjected regulation, associated with behaviors where external contingencies become internalized—though not yet accepted—as rules or demands that motivate and regulate one’s behaviors, and (6) identified regulation, associated with behaviors that are perceived as being a valuable, therefore, autonomously engaged. Finally, the third type of motivation is (7) amotivation, which is associated with a state where contingencies between actions and resulting outcomes are thought to be inexistent, leading to feelings of incompetence and helplessness, and ultimately, a state of complete absence of motivation. We employed the items in the original AMS [69]; the items were presented in a random order and not grouped by subscale. We employed an aggregate score of self-determination (Self-Determination Index, SDI) to serve as an overall measure of individual academic motivation. The SDI has been employed in previous motivation studies [62, 68], and is computed as a weighted average of the AMS subscales. The SDI has 4 components which are combined using the following weights: + 2 to the mean intrinsic motivation derived from the 3 intrinsic motivation scores, + 1 to the identified regulation score, − 1 to the mean between the external and introjected regulation scores, and − 2 to the amotivation score. Higher values of SDI indicate greater levels of self-determination in educational contexts. The English version of the AMS has acceptable levels of internal consistency (mean alpha = 0.81) and 4-week test–retest reliability (r = 0.79) [69]. The internal consistencies of the 7 subscales based on data from 86 Japanese university students ranged from alpha = 0.54 (Extrinsic motivation—Introjected regulation) to alpha = 0.83 (Intrinsic motivation to know) [8].

The AMS was collected to verify whether there were improvements in overall academic motivation (as measured by the SDI) following participation in a 2-week gratitude intervention. At the same time, the AMS allowed us to examine whether and how each one of the 3 academic motivation components, i.e., intrinsic motivation, extrinsic motivation and amotivation, was affected by the experimental manipulation.

*Personality Traits* The Neuroticism-Extraversion-Openness Inventory Five-Factor Inventory (NEO-FFI) [12], also known as the Big Five personality traits, consists of 60 items that serve to describe individual personality traits across 5 broad dimensions. The internal consistency of the 5 dimensions has been reported as ranging from alpha = 0.68 (Agreeableness) to alpha = 0.89 (Neuroticism) [12]. Results from a 3-month test–retest reliability were in the range of r = 0.75 (Agreeableness) to r = 0.83 (Conscientiousness) [12]. Similar results were observed in a 30-month test–retest reliability; coefficients were found tobe in the range from r = 0.73 (Agreeableness) to r = 0.86 (Openness) [45]. The Big Five Personality traits have been employed to predict academic outcomes [46, 51, 52] and are also associated with individual differences in academic motivation [38]. Here, we compared the NEO-FFI responses between the gratitude and control groups to ensure that there were no pre-existing differences regarding the Big Five personality traits that could have influenced the results of the 2-week experimental manipulation.

### Experimental schedule

All scales but the NEO-FFI were collected on 5 occasions during a period spanning 15 weeks, in total, to assess immediate and long-term effects associated with the 2-week gratitude journal intervention: Day0: pre-intervention assessment completed during the week immediately before the start of the intervention and before group assignment; Day7: mid-intervention assessment completed 6 days after its start; Day14: post-intervention assessment completed right after the end of the 2-week period; Day45: delayed follow-up assessment completed 1 month after the end of the intervention; Day105: delayed follow-up assessment completed 3 months after the end of the intervention. The NEO-FFI was collected only on Day0.

In the first 6 days of each week, participants in the gratitude group were asked to succinctly describe up to 5 events or thoughts that had led them to experience emotions associated with the state of being grateful. In addition to the gratitude journal task, participants were also requested to perform self-assessments regarding various aspects of their daily lives using visual analog ratings scales (implemented online as slidebars), namely, perceived level of stress, sleep duration, sleep quality, perceived level of happiness, extent of phone usage, and amount of face-to-face communication [42]. Participants were told that the middle point of each scale should be thought as the habitual level of the measured variable. Those values were recorded on a scale from 0 to 100, in increments of 0.1, but the numerical values were not displayed to the participants. Participants in the control group were asked to perform the same daily self-assessments but were exempted from the daily journal task. (This dataset was not further analyzed but the mean time courses of the ratings given by the participants in both groups can be found in Figures S1 and S2 in the Additional file 1) A video explaining how to interact with the system was made available online and participants were instructed to watch the video before engaging with the scheduled activities.

On the 7th day of each week, all participants were requested to answer the items in the initial questionnaire, with the exception of the NEO-FFI (GQ6/SWLS/PT/AMS). The intervention always started on a Monday, thus, Day7 and Day14 fell on a Sunday. Furthermore, follow-up questionnaires (GQ6/SWLS/PT/AMS) were sent via email 30 and 90 days after the last day of the study to all participants who completed the assessments on Day7 and Day14; participants were not told about the delayed follow-up assessments at recruiting time. Participants were monetarily rewarded for completing the tasks scheduled during the 2-week intervention (3000 yen), and in addition, for each completed follow-up questionnaire (1000 yen each). Participants were blind to the existence of different groups in the study and were not informed about the study hypotheses.

### Statistical analysis

All analyses were performed using SPSS version 24 (IBM, New York, USA) unless mentioned otherwise. To examine differences between groups, the dependent variable in question was analysed using a two-way repeated measures analysis of variance (rm-ANOVA). Degrees of freedom were adjusted using Greenhouse–Geisser estimates of sphericity (Mauchly’s sphericity test) whenever necessary. Statistical significance was defined at an alpha level less than 0.05. Post-hoc tests were performed when appropriate and adjusted for multiple comparisons using the Bonferroni correction.

## Results

### Participants

Four participants failed to complete the questionnaires scheduled on Day7 and Day14 and were dropped from the analysis (1 participant from the gratitude group and 3 participants from the control group), leading to an overall attrition rate of 4.8%. Because the online system allowed participants to access uncompleted tasks from previous days retroactively, we examined whether participants properly complied with the requirement of accessing the online system daily and performing the scheduled tasks on the same day. To accomplish that, we crosschecked the timestamps of the gratitude journal inputs collected on the first 6 days of each week (gratitude group), or the daily self-assessment of perceived level of happiness (control group), with the day the respective task was scheduled to be performed. Out of the 80 participants who completed the study, 40 participants (22 participants in the gratitude group and 18 participants in the control group) met the inclusion criterion of strictly following the experimental schedule in more than 2/3 of the 12 days a task was scheduled to be performed (average days off-schedule = 2.0, SD = 1.4). The excluded participants (19 participants in the gratitude group and 21 participants in the control group) were much less effective in keeping up with schedule (average days off-schedule = 8.0, SD = 2.7). For the sake of completeness, we performed the same analysis using the data from the entire sample (Full gratitude group, N = 41; Full control group, N = 39); results of the 2-week intervention regardless of schedule compliance are presented in the Additional file 1.

Without prior notice, all participants who had completed the 2-week intervention regardless of compliance with the experimental schedule were invited via email to answer the delayed follow-up questionnaire using the same online system. They were given 1 week to finish each questionnaire; 70 participants completed the 30-day follow-up (34 participants in the gratitude group and 36 participants in the control group) and the 90-day follow-up (36 participants in the gratitude group and 34 participants in the control group). Data regarding the composition of each group are summarized in Table 1.

**Table 1:** Number of participants by gender and mean age for each one of the groups

	Total	Female	Male	Mean Age (SD)
Recruited	84	35	49	21.75 (1.48)
Full control	42	17	25	21.67 (1.43)
Full gratitude	42	18	24	21.83 (1.54)
Failed to complete (control)	3	1	2	21.33 (1.15)
Failed to complete (gratitude)	1	1	0	22.00 (1.41)
Control	18	7	11	21.86 (1.53)
Gratitude	22	10	12	22.86 (1.33)
30-day follow-up respondents (full gratitude/full control)	70	32	38	21.64 (1.37)
90-day follow-up respondents (full gratitude/full control)	70	32	38	21.67 (1.38)
Control participants who responded to both follow-up’s	18	7	11	21.56 (1.34)
Gratitude participants who responded to both follow-up’s	20	10	10	22.00 (1.49)

*SD* standard deviation

### NEO-FFI

We first examined for latent differences in personality traits that may have existed at baseline by entering the mean scores of each one of the 5 NEO-FFI dimensions in a two-way rm-ANOVA with group (gratitude and control) as a between-subjects factor, and personality traits (Neuroticism, Extraversion, Openness, Agreeableness, and Conscientiousness) as within-subject factors.

Results revealed that there was a significant main effect of NEO-FFI traits (F(3.328, 126.481) = 8.668, *p* < 0.001), but no main effect of group (F(1, 38) = 0.404, *p* = 0.529) nor an interaction between the NEO-FFI traits and group (F(3.328, 126.481) = 1.633, *p* = 0.180). These results indicate that there were no substantial differences regarding NEO-FFI personality traits between participants in the gratitude and control groups that could have potentially influenced the results of the gratitude journal intervention. Mean scores for each one of the traits are shown in Table 2, by group.

**Table 2:** Mean scores of NEO-FFI traits by group

	N	E	O	A	C
Control (N = 18)	29.94 (8.79)	24.61 (5.78)	28.28 (7.50)	31.00 (3.20)	23.89 (6.28)
Gratitude (N = 22)	25.36 (6.83)	23.09 (5.22)	29.14 (6.45)	31.41 (6.64)	25.86 (5.27)

SD in parentheses

*N* neuroticism, *E* extraversion, *O* openness, *A* agreeableness, *C* conscientiousness

### Correlations between psychological scales

Before examining the responses to each psychological scale individually, we examined the relationships between scales by computing pairwise correlation coefficients (Pearson’s r) using the data collected on Day0, Day7 and Day14. Results for the gratitude and control groups—are summarized in Tables 3 and 4, respectively. (Results for the whole sample are shown in Additional file 1: Tables S3 and S4.)

**Table 3:** Correlation coefficients (Pearson’s r) between the scales collected on Day0, Day7 and Day14, from participants in the gratitude group

	SWLS	PT	AMS (SDI)
Day0			
GQ-6	0.269 (0.225)	0.167 (0.456)	− 0.322 (0.144)
SWLS	–	− 0.325 (0.140)	− 0.050 (0.825)
PT	–	–	0.261 (0.241)
Day7			
GQ-6	0.413 (0.056)	0.099 (0.661)	0.109 (0.630)
SWLS	–	0.074 (0.742)	− 0.046 (0.839)
PT	–	–	**0.505 (0.016)**
Day14			
GQ-6	**0.628 (0.002)**	0.227 (0.310)	0.136 (0.546)
SWLS	–	0.316 (0.152)	0.039 (0.862)
PT	–	–	**0.517 (0.014)**

*p* values in parentheses; values in boldface are significant at the *p* < 0.05 level

**Table 4:** Correlation coefficients (Pearson’s r) between the scales collected on Day0, Day7 and Day14, from participants in the control group

	SWLS	PT	AMS (SDI)
Day0			
GQ-6	0.088 (0.730)	0.346 (0.160)	0.247 (0.324)
SWLS	–	− 0.402 (0.098)	0.312 (0.207)
PT	–	–	0.327 (0.186)
Day7			
GQ-6	0.440 (0.068)	0.353 (0.151)	**0.483 (0.042)**
SWLS	–	− 0.281 (0.258)	**0.524 (0.025)**
PT	–	–	0.112 (0.657)
Day14			
GQ-6	**0.535 (0.022)**	0.454 (0.058)	0.383 (0.116)
SWLS	–	− 0.030 (0.906)	**0.591 (0.010)**
PT	–	–	0.133 (0.599)

*p* values in parentheses; values in boldface are significant at the *p* < 0.05 level

Consistent with our expectations, we found that none of the scales were correlated in the gratitude group (Table 3) or the control group (Table 4) at the onset of the study (Day0). Furthermore, results from the gratitude group suggested that a gradual progression took place during the 2-week intervention, from an initial state where none of scales were correlated (Day0), towards a state where the GQ-6 scores became positively correlated with the SWLS scores (r = 0.628), and the SDI scores became positively correlated with the PT scores (r = 0.517) on Day14.

Results from the control group showed that at the end of the intervention (Day14), the GQ-6 scores also became positively correlated with the SWLS scores (r = 0.535), much like the gratitude group. In addition, the SDI of the individuals in the control group became positively correlated with the SWLS (r = 0.591) on Day14. Because the GQ-6 scores in both the gratitude group and control group became correlated with the SWLS scores after the end of the 2 weeks, these results cannot be accounted to a specific effect associated with the performance of the gratitude journal activity. Rather, the simplest interpretation is that the emergence of such correlations is a result of study participation in general, regardless of condition. More interestingly, the correlation analysis results indicated that the SDI was distinctively associated with the measured psychological scales across groups; while in the absence of the gratitude activity the SDI became correlated with the SWLS score (control group), for the individuals who regularly engaged with the gratitude journal activity the SDI became correlated with the PT score, signaling that in the gratitude group, changes in academic motivation became associated with changes in perspective taking aptitude at the end of the intervention.

### GQ-6

In order to verify whether the 2-week gratitude journal intervention affected the individual gratitude disposition of study participants, we entered the GQ-6 scores in a two-way rm-ANOVA with group (gratitude and control) as a between-subjects factor and time (Day0, Day7, and Day14) as a within-subject factor.

Results failed to show any significant effect of group (F(1, 38) = 0.172, *p* = 0.681) or time (F(1.413, 53.689) = 1.743, *p* = 0.192). There was also no interaction between group and time (F(1.413, 53.689) = 0.232, *p* = 0.715).

### SWLS

To assess whether there were changes in individual well-being associated with engagement with the gratitude journal activity, we entered the SWLS scores in a two-way rm-ANOVA with group (gratitude and control) as a between-subjects factor and time (Day0, Day7, and Day14) as a within-subject factor.

**Fig. 1:**
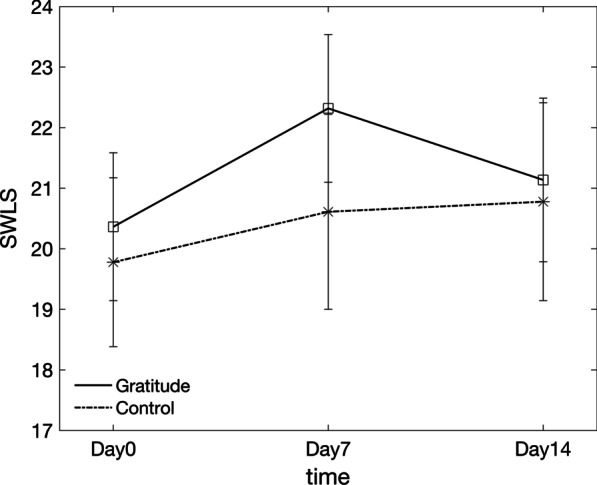
SWLS scores for the gratitude group and control group during the 2-week online gratitude journal intervention. Vertical bars show the standard error of the mean for each datapoint

Results showed that there was a significant main effect of time (F(2, 76) = 3.240, *p* = 0.045), though that was not accompanied by an effect of group (F(1, 38) = 0.223, *p* = 0.640) or an interaction between group and time (F(2, 76) = 0.848, *p* = 0.432). Post-hoc pairwise comparisons between the SWLS scores collected on different timepoints were not found to be significant (Day0 vs. Day7, *p* = 0.077; Day0 vs. Day14, *p* = 0.393; Day7 vs. Day14, *p* = 0.897). Results are summarized in Fig. 1.

### PT

We tested for differences in perspective taking aptitude resulting from the participation in the gratitude journal intervention by entering the PT scores in a two-way rm-ANOVA with group (gratitude and control) as a between-subjects factor and time (Day0, Day7, and Day14) as a within-subject factor. Results failed to detect significant effects for group (F(1, 38) = 0.127, *p* = 0.724), time (F(2, 76) = 2.189, *p* = 0.119) or an interaction between group and time (F(2, 76) = 0.669, *p* = 0.515).

### AMS (SDI)

To verify whether participation in the gratitude journal intervention positively impacted academic motivation, we entered the SDI scores derived from the AMS subscales in a two-way rm-ANOVA with group (gratitude and control) as a between-subjects factor and time (Day0, Day7, and Day14) as a within-subject factor.

**Fig. 2:**
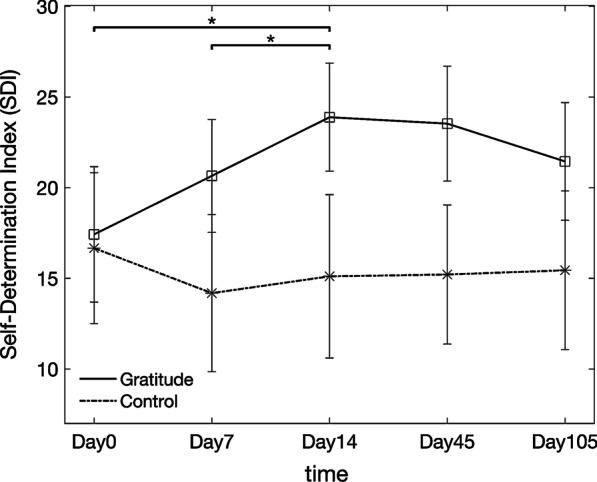
SDI for the gratitude group and control group during the 2-week online gratitude journal intervention and over the subsequent 3-month period (Day45: 1-month follow-up; Day105: 3-month follow-up). Asterisks indicate the pairwise comparisons between data collected from the gratitude group at different timepoints that were found to be statistically significant (*p* < 0.05, adjusted for multiple comparisons using Bonferroni correction). Vertical bars show the standard error of the mean for each datapoint

Results failed to detect effects for group (F(1, 38) = 1.139, *p* = 0.293) or time (F(1.312, 49.861) = 1.935, *p* = 0.167) but a significant interaction between group and time was observed (F(1.312, 49.861) = 4.714, *p* = 0.026). Because there was an interaction between group and time, we first performed a simple main effects analysis to assess whether there were differences in the SDI between groups at each timepoint but no significant differences were detected (Day0: F(1. 38) = 0.02, *p* = 0.890; Day7: F(1, 38) = 1.60, *p* = 0.213; Day14: F(1, 38) = 2.92, *p* = 0.096). Next, we examined whether there were differences in the SDI between different timepoints for each group; indeed, that was the case for the gratitude group (F(2, 76) = 6.42, *p* = 0.003) but not for the control group (F(2, 76) = 0.79, *p* = 0.457). Post-hoc tests using the gratitude group data showed that the SDI scores on Day14 (M = 23.89, SD = 13.32) were significantly higher than the scores on Day7 (M = 20.65, SD = 13.93, *p* = 0.006) and Day0 (M = 17.42, SD = 16.70, *p* = 0.008), even after applying the Bonferroni correction for multiple comparisons. The remaining pairwise comparison, Day7 versus Day0, failed to reach significance (*p* = 0.079). These results indicate that while the SDI scores for the control group did not change during the 2 weeks of the intervention, the SDI scores for the gratitude group gradually, and significantly, increased from Day0 to Day14. These results are summarized in Fig. 2 (Day0 to Day14).

#### SDI individual components

Having established that the SDI for the participants in the gratitude group, but not the control group, peaked on Day14, we sought to obtain further insights on how the observed enhancements in the aggregate score of academic motivation (SDI) related with the individual AMS subscales. To accomplish that, we conducted additional exploratory analyses to examine how the four components, namely, (1) the mean intrinsic motivation (derived from the 3 intrinsic motivation scores), (2) the identified regulation score, (3) the mean between the external regulation and introjected regulation score, and (4) the amotivation score, evolved throughout the course of the gratitude intervention period. Specifically, we entered the scores of the individual components of the gratitude group participants in a two-way rm-ANOVA with AMS component (intrinsic motivation, identified regulation, mean of external and introjected regulation, and amotivation) and time (Day0, Day7, and Day14) as within-subject factors, and examined whether there were significant effects for AMS component, for time or an interaction between both factors. Even though changes in SDI were not detected among control group participants, for the sake of completeness, we performed the same analysis using the scores from participants of the control group.

Results for the gratitude group showed that there was a significant effect of AMS component (F(3, 63) = 46.189, *p* < 0.001) but not time (F(1.509, 31.696) = 0.071, *p* = 0.884). In addition, that was accompanied by a significant interaction between AMS component and time (F(3.371, 70.797) = 4.090, *p* = 0.007). Given these results, we performed simple main effects analyses for each one of the AMS components with regards to time, followed by pairwise comparisons when necessary. We omitted the converse analysis, i.e., simple main effect analyses to examine differences between the AMS components for each point in time, because we judged that to be of relatively little interest.

From the equation used to compute the SDI, it is possible to see that an increase in either the mean intrinsic motivation score or the identified regulation score should result in an increase in the magnitude of the SDI, assuming all other components remain unchanged. In contrast, an increase in the mean value between the external regulation and introjected regulation scores, with all other things equal, should result in a decrease in the SDI. The simple main effects analyses showed that the results for the intrinsic motivation scores (F(2, 42) = 3.001, *p* = 0.061), identified regulation scores (F(2, 42) = 0.922, *p* = 0.406) and the mean of external and introjected regulation scores (F(2, 42) = 0.512, *p* = 0.603) were not significant, i.e., these scores did not change significantly through time, indicating that none of those components underlined the enhancements observed in the SDI of the individual in the gratitude group.

**Fig. 3:**
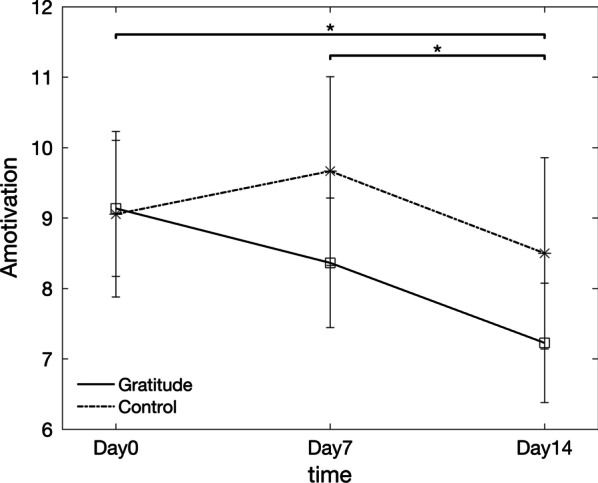
Amotivation score for the gratitude group and control group during the two-week online gratitude journal intervention. Asterisks indicate the pairwise comparisons between data collected from the gratitude group at different timepoints that were found to be statistically significant (*p* < 0.05, adjusted for multiple comparisons using Bonferroni correction). Vertical bars show the standard error of the mean for each datapoint

However, a significant effect of time was found in the amotivation scores (F(1.261, 26.484) = 6.231, *p* = 0.014). Pairwise comparisons indicated that the amotivation score on Day14 (M = 7.23, SD = 3.97) was significantly smaller than the score on Day7 (M = 8.36, SD = 4.31, *p* = 0.004) and the score on Day0 (M = 9.14, SD = 4.53, *p* = 0.038), adjusted for multiple comparisons using Bonferroni correction. Decreases in amotivation, with all other things equal, should lead to improvements of an individual’s SDI. Given that no effects were found in the data from the other AMS components, these results indicate that the enhancement observed in the SDI of individuals in the gratitude group was a direct result of decreases in amotivation levels resulting from the regular engagement with the gratitude journal activity. No significant difference was found between the Day0 and Day7 scores (*p* = 0.524). Results are summarized in Fig. 3.

Results from the analysis performed using the scores from the control group showed that there was a significant effect of AMS components (F(1.834, 31.170) = 19.788, *p* < 0.001) but not for time (F(2, 34) = 0.059, *p* = 0.942) nor an interaction between both factors (F(3.229, 54.892) = 2.006, *p* = 0.119). We did not perform the simple main effect analyses for each point in time with regards to the AMS components for the same reason mentioned above. (Figures S3, S4, S5 and S6 in the Additional file 1 show the time courses for the intrinsic motivation, identified regulation, external regulation and introjected regulations scores, respectively.)

Taken together, these results indicate that the enhancements observed in the SDI of participants in the gratitude group were primarily driven by a gradual decrease in the amotivation scores of individuals who engaged with the daily gratitude journal activity. Such effect was not present in the data from the control group.

#### Delayed follow-ups

Because the SDI of participants in the gratitude group peaked on Day14, we sought to examine whether the gains in academic motivation observed among participants in the gratitude group receded 1 and 3 months after the end of the intervention. To assess that, we entered the SDI scores collected on Day14, Day45 and Day105 in a one-way rm-ANOVA with time as a within-subject factor. No significant effect was detected for time (F(2, 38) = 1.354, *p* = 0.270), indicating that even 3 months after the end of the gratitude journal intervention, there were still no significant signs that the SDI scores of participants in the gratitude group had dropped from their post-intervention levels at Day14. These results indicate that the positive impact of regularly engaging with a gratitude journal activity outlasted by far the duration of the intervention itself. Results are summarized in Fig. 2 (Day14 to Day105).

#### Gratitude journal contents

Finally, we examined the entries in the gratitude journal that were inputted online by the participants in the gratitude group. Participants entered on average 2.1 entries per day (SD = 1.2, range from 1 to 5). Across participants, the number of entries was significantly correlated with the mean GQ-6 score of Day0, Day7 and Day14 (Pearson’s r = 0.439, *p* = 0.041), indicating that individuals with overall higher mean GQ-6 scores tended to list up more events or thoughts associated with the state of being grateful. We then pooled the text data from all entries and parsed them using MeCab [39]. Table 5 shows the 10 most frequently occurring nouns in the journals kept by the participants in the gratitude group. Though looking at the nouns independently and detached from the original context only allow a limited view of the connotation that was intended to be communicated by the participants, judging from the most frequently occurring nouns at face value, it does not seem to be the case that participants were particularly mindful of academic activities or campus life when formulating the entries to the gratitude journal. Rather, the entries seem to describe stereotypical situations that usually lead people to experience feelings of gratitude, for instance, when one receives the help from a senior student or co-worker in the context of a part-time job, or when one feels grateful for an act of kindness performed by a relative, e.g., mother, or a friend.

**Table 5:** The 10 most frequently occurring nouns in the gratitude journal entries of the participants in the gratitude group with the respective number of occurrences

Noun	Number of occurrences
Friend	84
Mother	48
Senior student or co-worker	37
Part-time job	29
Meal	29
Me/myself	25
Weather	17
Parents	14
Rain	13
Shop clerk	12

The total number of nouns was 964

## Discussion

Experimental work examining the effects of gratitude intervention on students’ academic motivation is still very limited and the reported results are so far mixed. To the best of our knowledge, the only study in the gratitude research literature that focused specifically on the relationship between a gratitude intervention and the academic motivation of university students was not able to detect significant motivational improvements [37], likely due to the relative low intensity of the employed gratitude activity. To clarify the benefits that a gratitude intervention can have on the academic motivation of university students, we performed a 2-week gratitude intervention, during which participants were requested to engage daily with a gratitude journal activity. Such level of intensity is more comparable with studies that have previously succeeded in detecting significant gratitude effects, e.g., [23]. Participants were randomly assigned to either an active manipulation group (gratitude group) or a neutral control condition group (control group). Comparisons between pre-intervention (collected before group assignment) and post-intervention (collected immediately after the end of the intervention) assessments revealed, first and foremost, that the aggregate score of academic motivation (SDI) improved only among participants in the gratitude group. Interestingly, that effect was only observed in the participants who met the inclusion criteria of complying with the daily experimental schedule, i.e., only the students who regularly engaged with the gratitude journal during the 2 weeks of the intervention enjoyed the motivational enhancements. This highlights the importance of tuning the parameters of a gratitude intervention, i.e., the activity type, its execution frequency as well as the overall intervention duration, so that the resulting intensity maximizes the occurrence of positive effects. Excessive engagement with a gratitude activity is likely to lead to adverse effects in the opposite direction [32]; because different populations might respond differently to the same manipulation, a more systematic investigation needs to be performed to clearly establish how the intensity of an intervention relates with the positive outcomes reported in the gratitude research literature.

Most remarkably, the results from the delayed follow-up questionnaires revealed that even 3 months after the end of the 2-week intervention there were no signs that the enhancements observed in the SDI of the gratitude group had drifted away significantly, suggesting that the benefits acquired from the gratitude journal intervention reverberated well past the duration of the manipulation itself.

Inspection of the contents entered in the gratitude journal did not reveal any obvious sign that students were deliberately mindful of academic activities or any other aspect related to school during the 2 weeks of the intervention, which would open the possibility that the observed enhancements were a trivial and direct by product of the journaling activity itself. Rather, judging from the list of most commonly occurring nouns, participants often described typical ‘gratitude’ situations, e.g., as receivers of assistance from friends and relatives. Engaging in thoughts associated with typical grateful events seems to be the driver of the enhancements observed in terms of academic motivation immediately after the intervention.

The SDI is derived from the subscales of the AMS [69]; further analyses revealed that the enhancements observed in the SDI of participants in the gratitude group was driven by a gradual decrease in the amotivation scores. Amotivation is thought to be at the lowest end of the motivation construct continuum among all subscales of the AMS. Though the construct of amotivation itself is likely to be multidimensional [41], in school contexts high amotivation basically reflects a lack of motivation toward academic activities that originates from the perception that one’s behavior, as well as the outcomes that follow, are solely caused by external contingencies that are beyond one’s control, likely culminating in increased feelings of incompetence and helplessness, if left unattended. Higher levels of amotivation are associated with greater perceived stress, poorer adjustment to university life and higher levels of psychological distress [4]. Amotivation was also found to be negatively correlated with a student’s average grade, as well as the degree of commitment a student feels towards the university [3], and positively correlated with Anxiety and Depression levels, as measured by the General Health Questionnaire [3]. Interestingly, GQ-6 scores were found to be negatively related to amotivation and positively related to autonomous and controlled motivation in a population of high-school students [66]. Given the broad reach of the detrimental influence of amotivation, gratitude interventions, alongside classroom-based interventions [11] and other psychological interventions [55], may prove to be an effective and relatively simple device to enhance student engagement and positively impact the overall mental health of university students.

What could be the mechanism linking an activity that regularly reminds people to recollect personal experiences associated with the emotion of gratitude—increasing their awareness to such experiences during and perhaps beyond the period of the intervention—to the observed enhancements in academic motivation, specifically, the reduction in amotivation levels? One possibility is that making people more aware of daily life experiences that made them feel grateful results in an increased appreciation to the “blessings” endowed by external agents, which happens to be the hallmark of the gratitude experience. That possibly led participants in the gratitude group to reassess the circumstances they presently enjoy, including the fact that they are enrolled in an institution of higher education, resulting in an improved sense of purpose towards the activities they engage with, most saliently though not limited to, academic activities. The enhanced sense of purpose may have co-occurred with an improved sense of appreciation to the circumstances they currently enjoy, and the realization that they are somehow better off than their peers, leading to an enhanced drive (motivation) to make the most of the opportunities currently at hand, which here took the form of enhancements in SDI among gratitude group participants. Even though we did not detect improvements in perspective taking aptitude as measured with the PT in the gratitude group as initially hypothesized, it is indicative that after the end of the 2 weeks (Day14), PT scores were significantly correlated with the SDI scores of the gratitude group participants but not participants in the control group (whose SDI scores became correlated with the SWLS scores). This result indicates that participants in the gratitude group who experienced greater improvements in terms of SDI score also displayed greater increases in PT scores. The PT was originally designed to measure the facet of the construct of empathy that is centered in the aptitude of seeing things from the perspective of other people, which in effect amounts to measuring one’s ability to step “outside the self”, as stated by the author of the scale [15], and examine the world from a less subjective frame of mind. Though the PT and SDI were correlated at the end of the intervention, the exact mechanism that led to such effect remains to be verified, i.e., does engaging with a daily gratitude journal activity lead to changes in perspective taking aptitude, which in its turn causes the enhancement in overall SDI? Or is the strengthened correlation caused by a distinct chain of events?

This study has a few limitations. One caveat to keep in mind is that participants in the control group were not assigned a comparable active task component (e.g., “list up the habitual activities you performed during the day”), though in all other respects both groups were identical. Further research is necessary to clarify whether the observed enhancements in academic motivation constitute a specific product of the gratitude journal manipulation or whether similar results can be obtained by other types of intervention that arguably tap into positive emotions [7], e.g., keeping a journal of positive daily life events, which have shown to produce similar effects to those yielded by gratitude interventions with regard to typically measured outcome variables associated with well-being and mood [19].

One important finding derived from the current results is that proper engagement with the daily gratitude journaling task seems to be a necessary condition for the motivation benefits to materialize; enhancements in SDI were detected in the gratitude group but not when using the dataset that included data from participants who did not strictly comply with the experimental schedule (Full gratitude group, Additional file 1). Monitoring proper task attendance was possible because all data was collected electronically via an online web system. Though there were no obvious differences in terms of NEO-FFI personality traits between the compliant and non-compliant cohorts, it remains to be verified whether the same benefits will be observed in individuals who do not spontaneously stick to the recommendation of making daily entries to their gratitude journals but only do so after being prompted to.

It is also important to attend to the point that participants in this study were all Japanese university students. Individuals from Asian cultural backgrounds are thought to hold different views regarding the balance between individualism and collectivism, compared to individuals of Western cultural backgrounds [34]. Asian societies are typically assumed to attribute greater value to conformity to norms and the interdependence among in-group individuals than Western societies; such attributes likely influence individual motivational processes as well [43]. Cultural differences may also account for how individuals from Asian societies regard and express affective states such as happiness [65] and gratitude [10]. These discrepancies limit the generalizability of the current results until future studies investigate the extent to which gratitude interventions can enhance the motivation of students from different cultural backgrounds.

Related to the above, one potential problem with the Japanese version of the AMS is that two of the 7 subscales were reported to have consistency alpha scores below the usually accepted threshold of 0.70 [61], i.e., alpha = 0.54 (Extrinsic motivation—Introjected regulation) and alpha = 0.69 (Extrinsic motivation—external regulation) [8]. Though no effects were observed regarding the subscales associated with extrinsic motivation in our results, it is possible that the low reliability might be reflecting a fundamental dissonance between Japanese individuals and the facets of extrinsic motivation probed by the AMS. In a broader sense, this highlights the importance of taking into account cultural factors in motivation and gratitude studies.

Another limitation of the current study is that we were unable to detect changes in the GQ-6 and SWLS scores that were specific to the participants of the gratitude group. The GQ-6 was the only metric directly associated with the target of the manipulation. The GQ-6 is often considered to be a trait measure of gratitude disposition, e.g., [73], and thus, arguably less prone to alterations over relatively short periods of time. Largely in line with that, the effect of gratitude interventions is much smaller on measures of grateful disposition than on measures of grateful mood across studies [19]. Still, in order to clarify the mechanism underlying the effects observed in this study, future research must attempt to clearly verify whether and how gratitude journal interventions can effectively affect parameters associated with the perception of, or proneness to experience the emotion of gratitude in daily life situations.

Data collection in this study was performed online, which greatly facilitated the analysis of the gratitude journal contents. Employing more advanced natural language processing techniques will allow a systematic inspection of real-life gratitude evoking situations, helping unravel, for instance, when and owing to whom people most commonly experience gratitude. Such a detailed qualitative and quantitative characterization of the circumstances revolving the emotion of gratitude will help delineate a mechanistic model of that emotion that will serve to optimize future interventions.

Finally, it still remains an open question whether the effect of gratitude interventions extends beyond the domain of self-report scales, and can ultimately influence actual student learning behaviors, and their perception and attitude towards academic activities in a positive way. Students’ grades have been commonly employed as a metric to assess real-life effects resulting from gratitude interventions. However, results so far have been somewhat mixed [2, 30]. One possibility is that grades are a narrow window to assess the impact of gratitude interventions. Future studies will have to employ broader, more multi-faceted definitions and measures of academic performance to fully unravel the benefits that gratitude interventions have on students’ academic lives.

## Conclusions

Academic motivation is thought to be a primary determinant of academic achievement and overall satisfaction with school activities. For that reason, the development of interventions that effectively improve students’ motivation in school settings has been regarded as a critical issue to foster student growth [53, 71]. The current findings show that a relatively simple online gratitude intervention can have a positive impact on the academic motivation of university students, and most importantly, that such effects may be long lasting. Online interventions have the  advantage of being more accessible, scalable and affordable to large portions of the population. Building a solid evidence base to support their deployment will be essential to unleash their true potential in the future.

## Supplementary Information

**Additional file 1.** Time courses of the daily self-assessments given by the gratitude and control group participants, results from the analysis of data from the entire sample of participants regardless of schedule compliance, and time courses of the intrinsic motivation, identified regulation, external regulation and introjected regulation scores.

## Data Availability

The datasets generated and analyzed during the current study are available from the corresponding author upon reasonable request.

## Abbreviations

AMS: Academic Motivation Scale
GQ-6: Gratitude Questionnaire
SWLS: Satisfaction with Life Scale
PT: Perspective Taking Scale
NEO-FFI: Neuroticism-Extraversion-Openness Inventory Five-Factor Inventory
SDI: Self-Determination Index
SD: Standard deviation
rm-ANOVA: Repeated measures analysis of variance
