# Supplementary material for: Enhanced academic motivation in university students following a 2-week online gratitude journal intervention
Source: BMC Psychol. 2021 May 13;9:71. doi: 10.1186/s40359-021-00559-w (PMC8117657; doi:10.1186/s40359-021-00559-w)

Supplementary Material

Enhanced Academic Motivation in University Students Following a Two-week Online Gratitude Journal Intervention


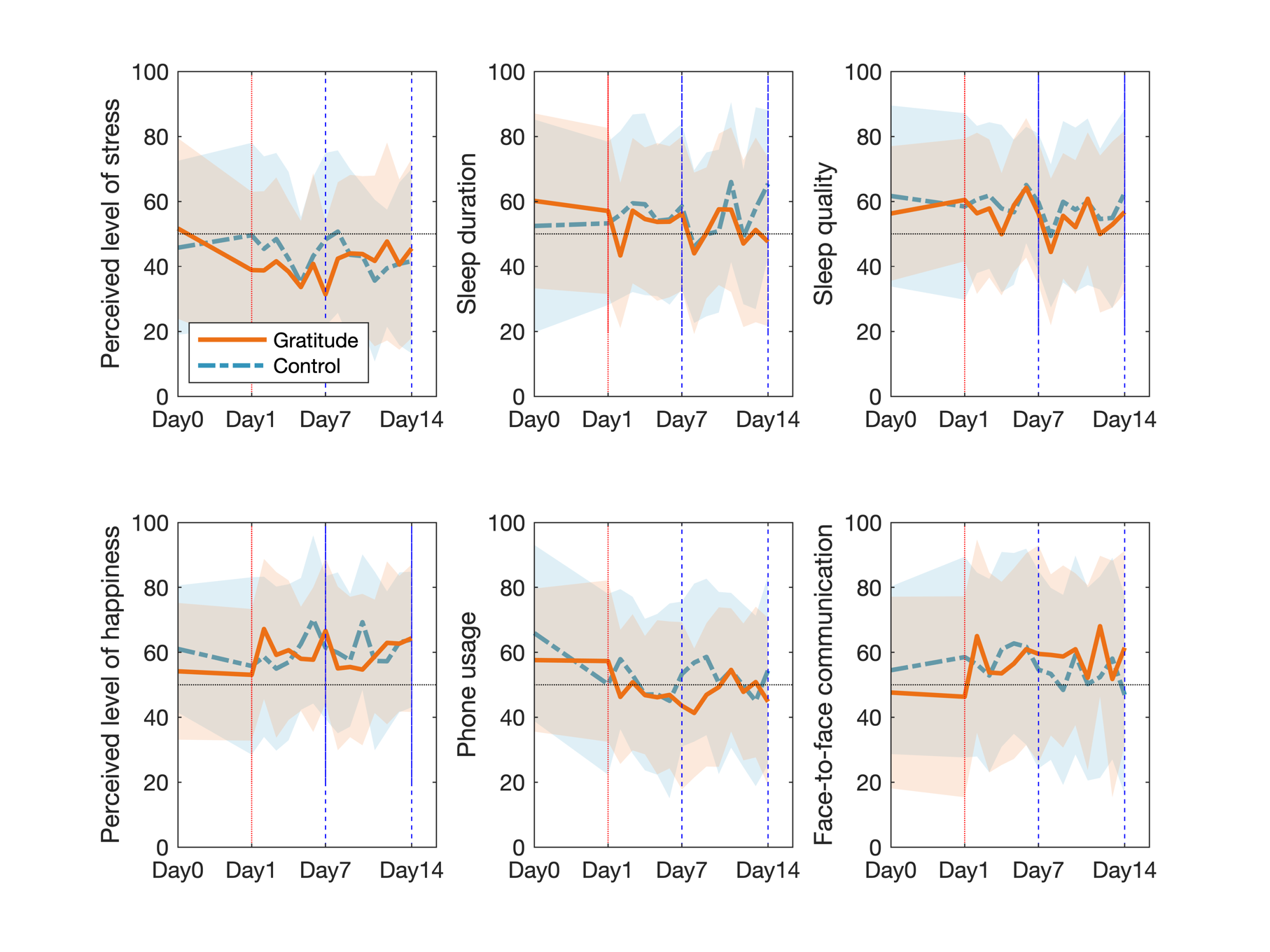
Figure S1. Mean ratings of the daily self-assessments performed by participants in the gratitude and control groups using a scale from 0 to 100. Top panels: Perceived level of stress (left), sleep duration (center), sleep quality (right). Bottom panels: Perceived level of happiness (left), phone usage (center), amount of face-to-face communication (left). The shaded area shows the standard deviation of each group sample. Participants provided the data corresponding to Day0 sometime on the week before the start of the intervention.

# Results for the Full Gratitude and Full Control Groups


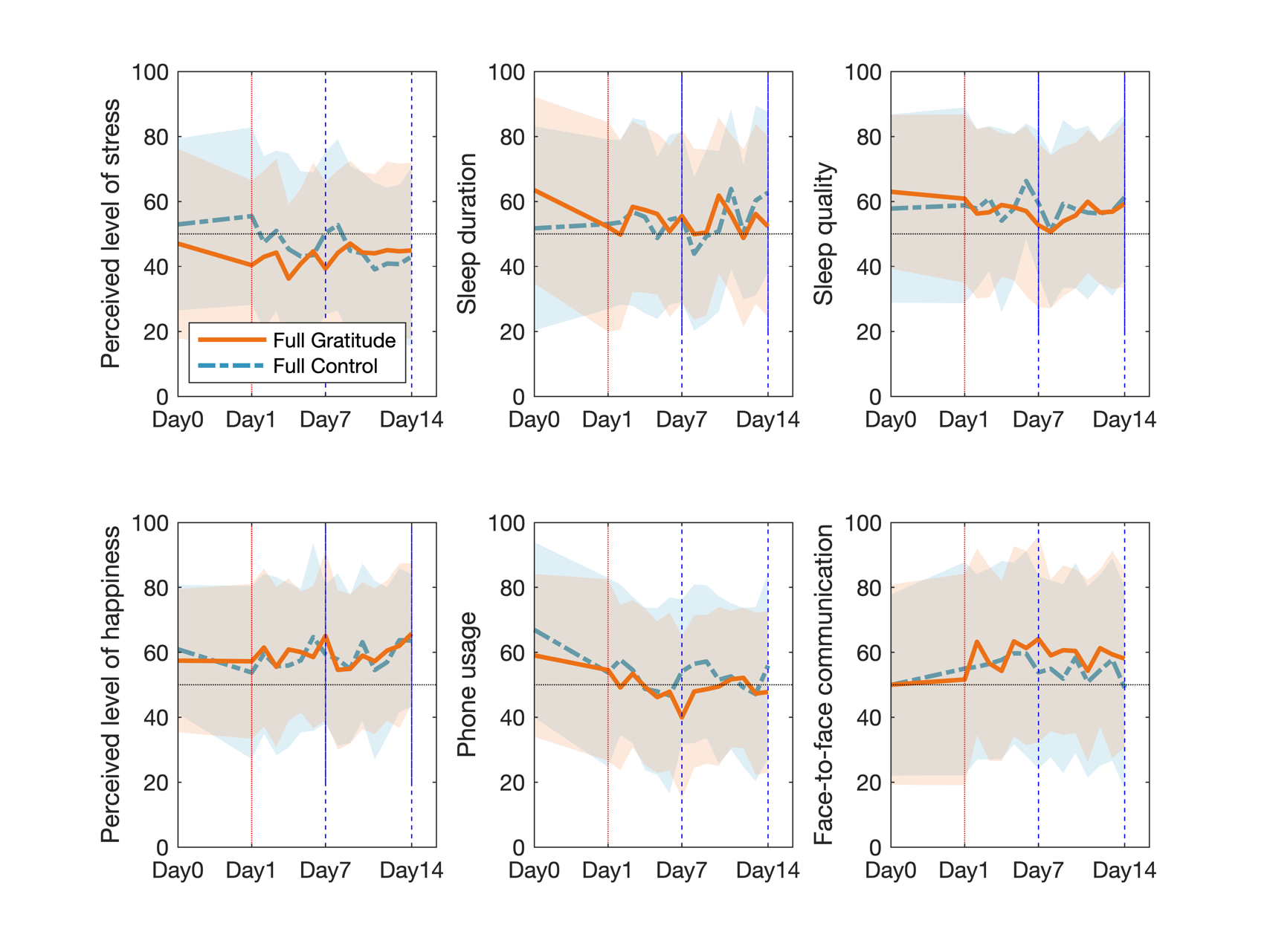
Figure S2. Mean ratings of the daily self-assessments performed by participants in the Full Gratitude and Full Control groups. Panels are as described in the caption of Figure S1.

# Results

The same statistical analyses were performed using data from the entire sample of 80 participants regardless of compliance with the experimental schedule, i.e., Full gratitude (N = 41) and Full control (N = 39) groups.

***NEO-FFI***

Results revealed a significant main effect of NEO-FFI traits (F(3.323, 259.191) = 11.971, p < 0.001), but did not detect a main effect of group (F(1, 78) = 0.573, p = 0.451). The interaction between NEO-FFI traits and group (F(3.323, 259.191) = 1.603), p = 0.184) was also not found to be statistically significant. Mean scores for each one of the traits are shown in Table S2, by group.

We also assessed whether differences in compliance could be attributed to differences in personality traits between the two subgroups. Data from the compliant participants in the gratitude and control groups of the original analysis were combined into a single group (Compliant group) and compared with the data from the rest of the participants (Non-Compliant group). No differences between the Compliant and Non-Compliant groups were detected; though there was a significant main effect of NEO-FFI traits (F(3.247, 253.248) = 11.837, p < 0.001), there was no main effect of group (F(1, 78) = 0.785, p = 0.378) nor an interaction between the NEO-FFI traits and group (F(3.247, 253.248) = 1.269, p = 0.285).

These results indicate that there were no substantial differences regarding NEO-FFI personality traits between the Full gratitude and Full control groups, nor a difference between compliant and non-compliant participants.

**GQ-6**

Results from a two-way rm-ANOVA using the GQ-6 responses from the Full gratitude and Full control groups did not indicate the existence of an effect of group (F(1, 78) = 0.169, p = 0.682) or time (F(1.693, 132.042) = 0.971, p = 0.369), or an interaction between group and time (F(1.693, 132.042) = 2.053, p = 0.140).

**SWLS**

Results from a two-way rm-ANOVA using the SWLS responses from the whole sample did not indicate an effect of group (F(1, 78) = 2.762, p = 0.101) or time (F(2, 156) = 0.451, p = 0.638), nor the existence of an interaction between group and time (F(2, 156) = 1.029, p = 0.360).

**PT**

Results from a two-way rm-ANOVA using the PT responses from the whole sample indicated that there were no effects for group (F(1, 78) = 0.094, p = 0.760). Effects for time (F(2, 156) = 2.817, p = 0.063) and the interaction between group and time (F(2, 156) = 2.939, p = 0.056) were also not significant, albeit in much less unequivocal levels.

**AMS (SDI)**

Results from a two-way rm-ANOVA using the SDI scores from the whole sample showed no signs of effects for group (F(1, 78) = 1.310, p = 0.256), time (F(1.611, 125.620) = 0.435, p = 0.605) or an interaction between group and time (F(1.611, 125.620) = 3.042, p = 0.062), though the results for the latter were much less unequivocal compared to the former two.

Table S2. Mean scores of NEO-FFI traits for the Full control and Full gratitude groups (SD in parentheses). N: Neuroticism; E: Extraversion; O: Openness; A: Agreeableness; C: Conscientiousness. ^*^Participants who did not complete the intervention were excluded.

|  | N | E | O | A | C |
| --- | --- | --- | --- | --- | --- |
| Full Control group^*^ (N = 39) | 29.95 (7.58) | 24.31 (6.98) | 30.02 (7.03) | 30.00 (5.50) | 24.59 (5.92) |
| Full Gratitude group^*^ (N = 41) | 26.61 (7.15) | 24.39 (6.43) | 29.02 (5.68) | 30.12 (6.68) | 26.24 (6.08) |

Table S3: Correlation coefficients (Pearson’s r) between the assessments collected on Day0, Day7 and Day14, from the participants of the Full gratitude group (p-values in parentheses; values in boldface are significant at the p < 0.05 level).

Day0

|  | SWLS | PT | AMS (SDI) |
| --- | --- | --- | --- |
| GQ-6 | 0.184 (0.248) | 0.262 (0.097) | -0.004 (0.978) |
| SWLS | - | 0.032 (0.844) | 0.203 (0.202) |
| PT | - | - | **0.361 (0.020)** |

Day7

|  | SWLS | PT | AMS (SDI) |
| --- | --- | --- | --- |
| GQ-6 | 0.257 (0.104) | 0.103 (0.521) | 0.075 (0.642) |
| SWLS | - | -0.007 (0.966) | 0.174 (0.276) |
| PT | - | - | **0.348 (0.026)** |

Day14

|  | SWLS | PT | AMS (SDI) |
| --- | --- | --- | --- |
| GQ-6 | **0.428 (0.005)** | 0.292 (0.064) | 0.013 (0.933) |
| SWLS | - | 0.240 (0.131) | 0.197 (0.217) |
| PT | - | - | **0.315 (0.045)** |

Table S4: Correlation coefficients (Pearson’s r) between the assessments collected on Day0, Day7 and Day14, from the participants of the Full control group (p-values in parentheses; values in boldface are significant at the p < 0.05 level).

Day0

|  | SWLS | PT | AMS (SDI) |
| --- | --- | --- | --- |
| GQ-6 | 0.297 (0.066) | 0.110 (0.506) | 0.144 (0.382) |
| SWLS | - | -0.193 (0.240) | **0.422 (0.007)** |
| PT | - | - | 0.133 (0.419) |

Day7

|  | SWLS | PT | AMS (SDI) |
| --- | --- | --- | --- |
| GQ-6 | **0.486 (0.002)** | 0.172 (0.295) | **0.318 (0.048)** |
| SWLS | - | -0.046 (0.779) | **0.517 (0.001)** |
| PT | - | - | 0.071 (0.668) |

Day14

|  | SWLS | PT | AMS (SDI) |
| --- | --- | --- | --- |
| GQ-6 | **0.625 (0.000)** | **0.334 (0.038)** | **0.499 (0.001)** |
| SWLS | - | 0.094 (0.570) | **0.561 (0.000)** |
| PT | - | - | 0.269 (0. 098) |

Figure S3. Mean intrinsic motivation for the gratitude and control groups during the two-week online gratitude journal intervention. Vertical bars show the standard error of the mean for each datapoint.


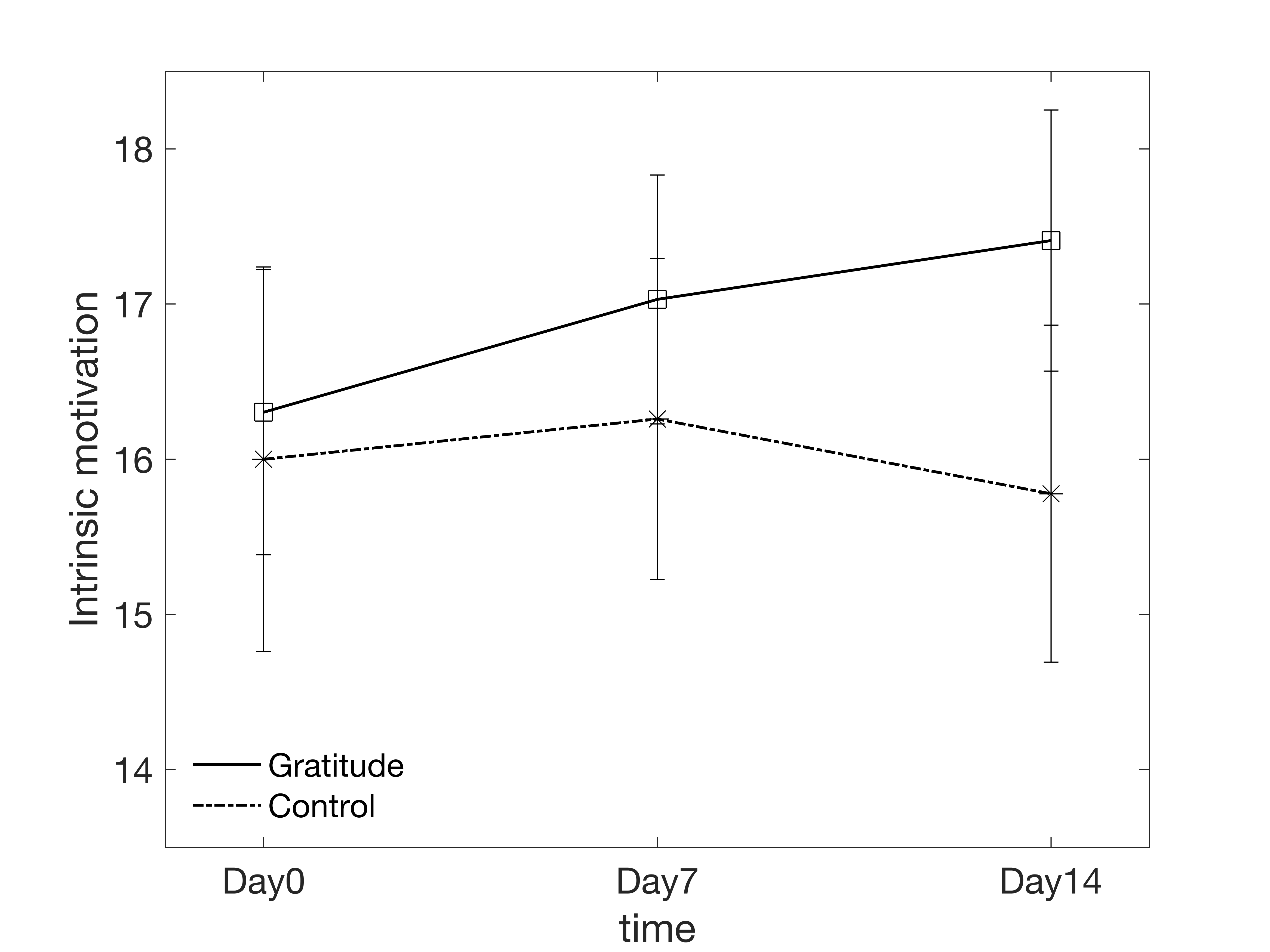


Figure S4. Identified regulation scores for the gratitude and control groups during the two-week online gratitude journal intervention. Vertical bars show the standard error of the mean for each datapoint.


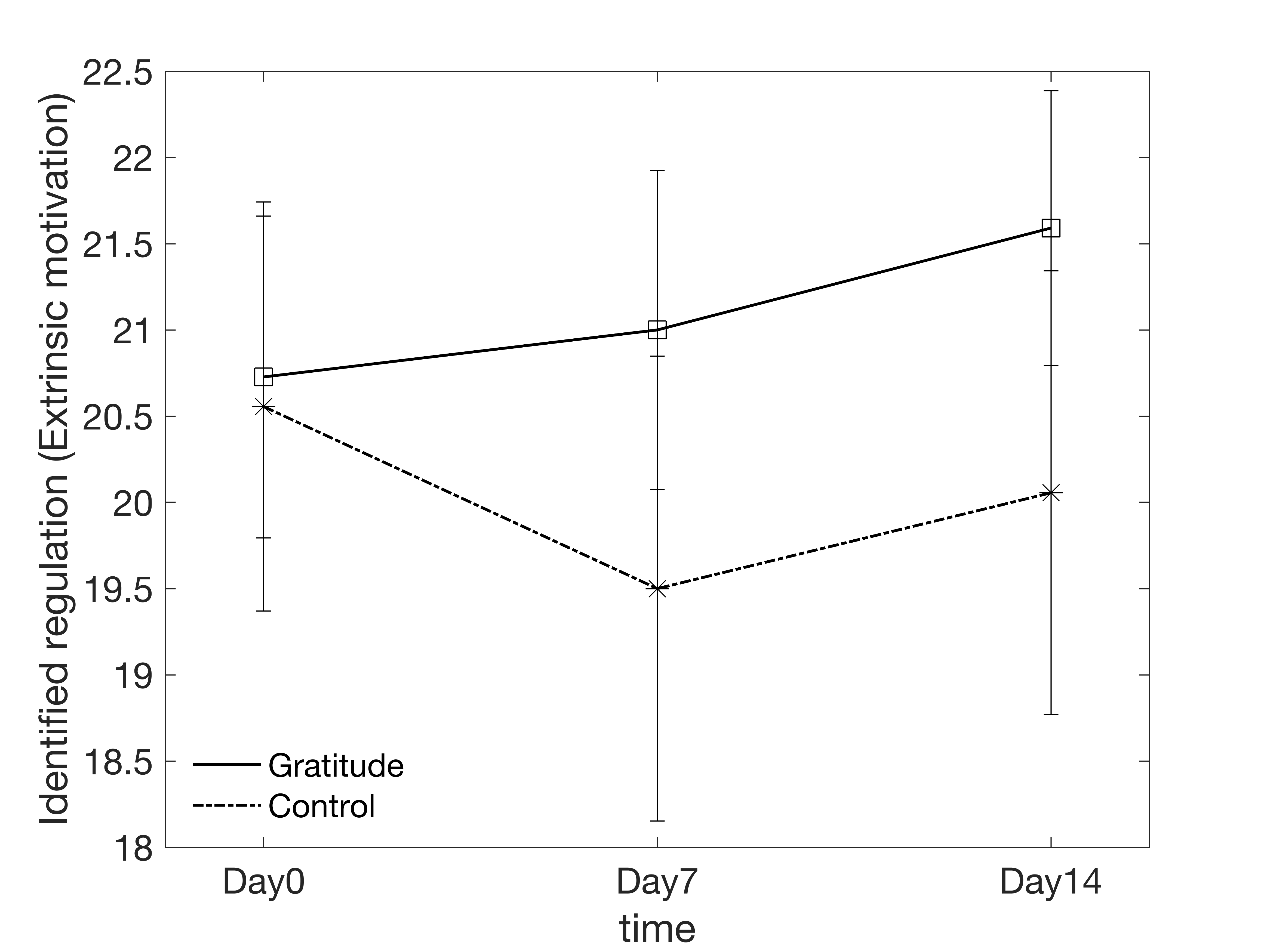


Figure S5. External regulation scores for the gratitude and control groups during the two-week online gratitude journal intervention. Vertical bars show the standard error of the mean for each datapoint.


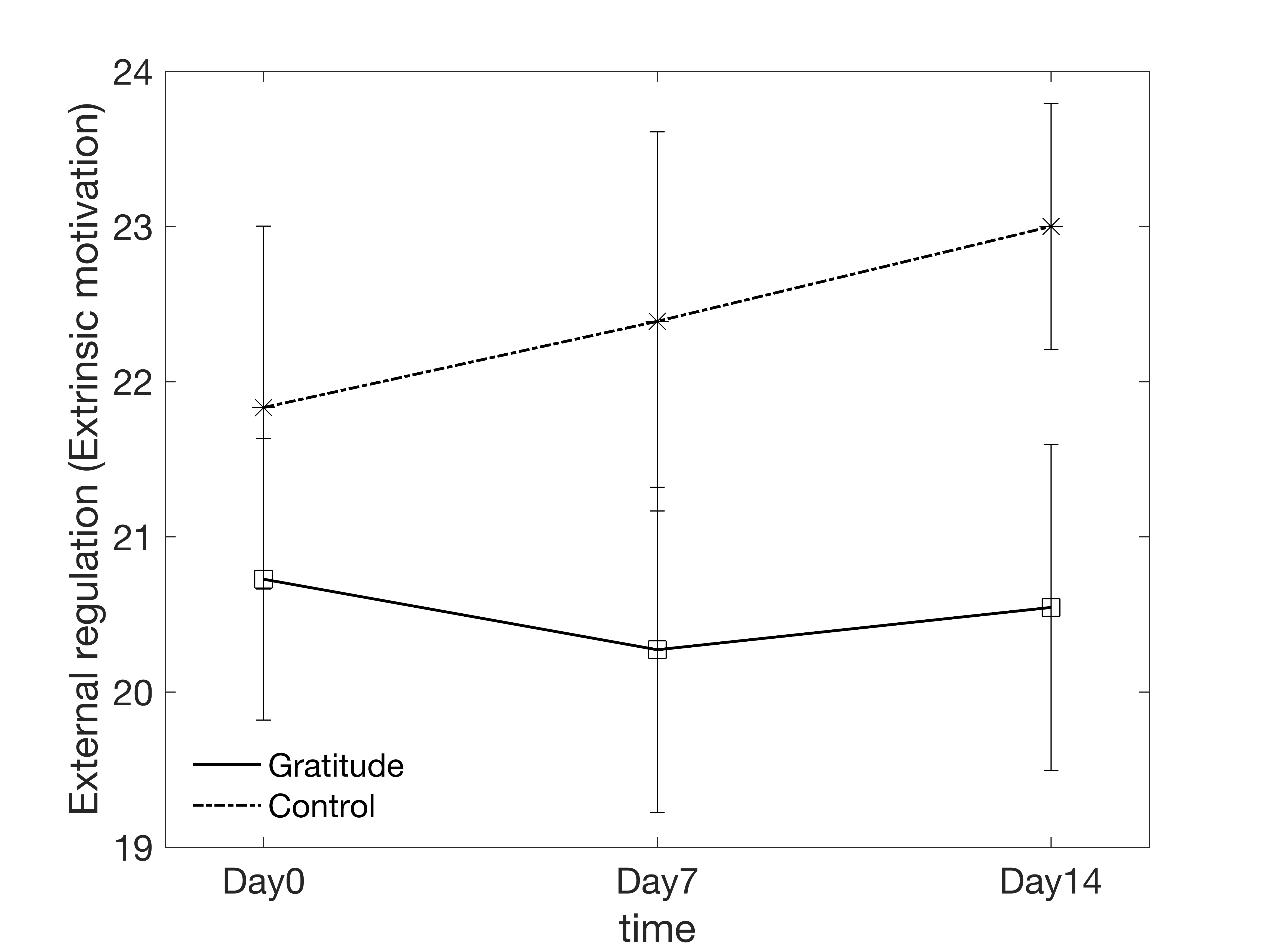


Figure S6. Introjected regulation scores for the gratitude and control groups during the two-week online gratitude journal intervention. Vertical bars show the standard error of the mean for each datapoint.


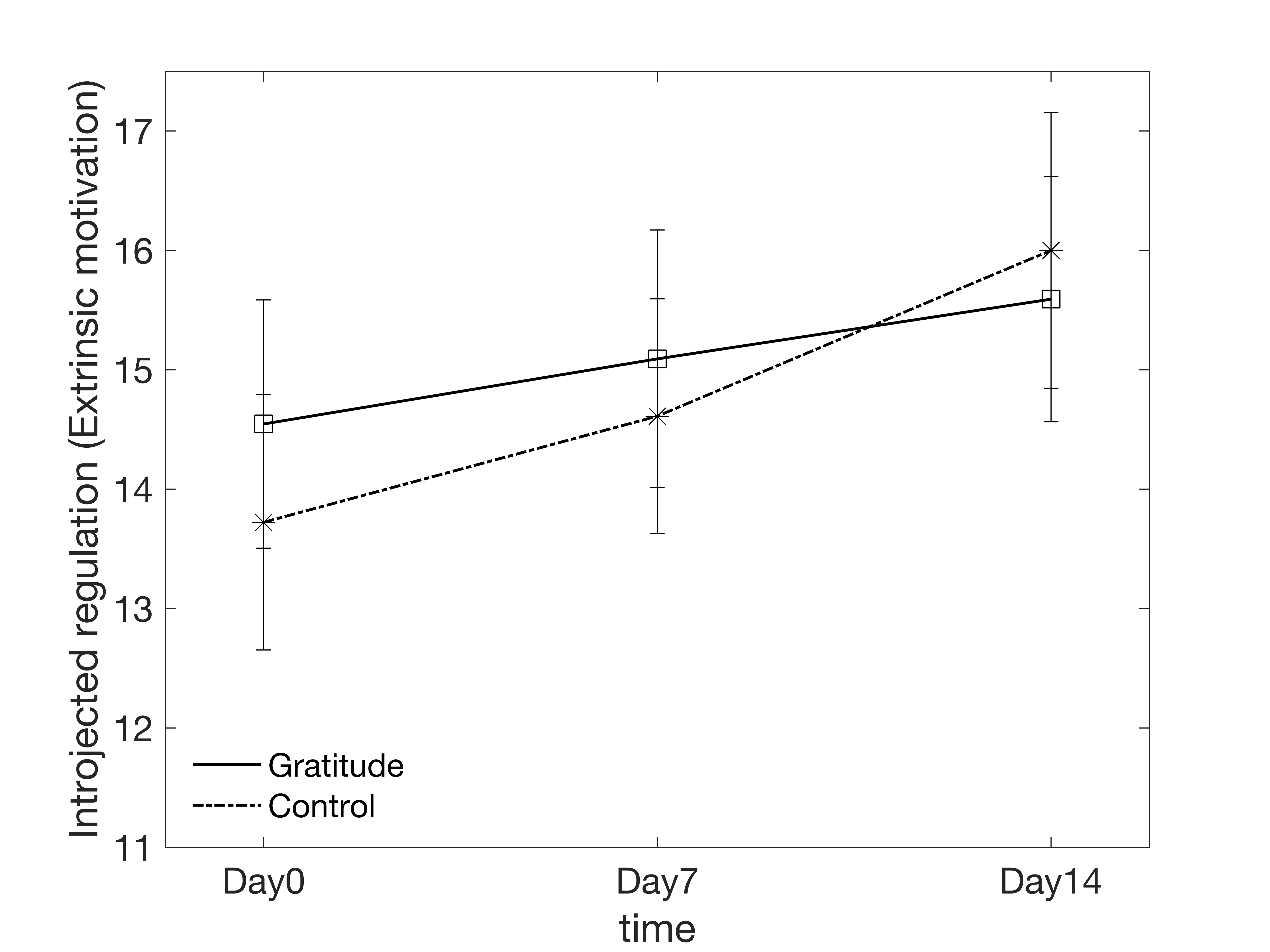

Supplement: Supplementary file 1 — Additional file 1. Time courses of the daily self-assessments given by the gratitude and control group participants, results from the analysis of data from the entire sample of participants regardless of schedule compliance, and time courses of the intrinsic motivation, identified regulation, external regulation and introjected regulation scores. [file 40359_2021_559_MOESM1_ESM.docx]
